# Supplementary material for: ABEL-FRET bridges the timescale gap in single-molecule measurements of the structural dynamics in the A2A adenosine receptor
Source: Commun Chem. 2026 Mar 9;9:114. doi: 10.1038/s42004-026-01941-8 (PMC12972066; doi:10.1038/s42004-026-01941-8)
Supplement: Supplementary file 1 — Supplementary Material [file 42004_2026_1941_MOESM1_ESM.pdf]

Supplementary information

“ABEL-FRET bridges the timescale gap in single-molecule measurements of the structural dynamics in the A_{2A} adenosine receptor”

Maslov et al.

Contents:

Supplementary figure 1. Characterization of the $A_{2A}AR_{L225C/Q310C}$ reconstituted in lipid nanodiscs.
Supplementary figure 2. The distribution of diffusion coefficients of $A_{2A}AR$ in lipid nanodiscs.
Supplementary figure 3. Analysis of fluorescence intensities of individual apo and ligand-bound $A_{2A}AR$ molecules.
Supplementary table 1. Fitting parameters of fluorescence intensity distributions for apo and ligand-bound $A_{2A}AR$.
Supplementary figure 4. The correlation functions for the fluorescence intensities of donor and acceptor fluorophores in apo and ligand-bound $A_{2A}AR$.
Supplementary figure 5. Normalized intensity autocorrelation function calculated from a 5-min time-correlated single photon counting (TCSPC) recording of the labeled apo $A_{2A}AR$ in lipid nanodiscs in the working ABEL trap.
Supplementary references.

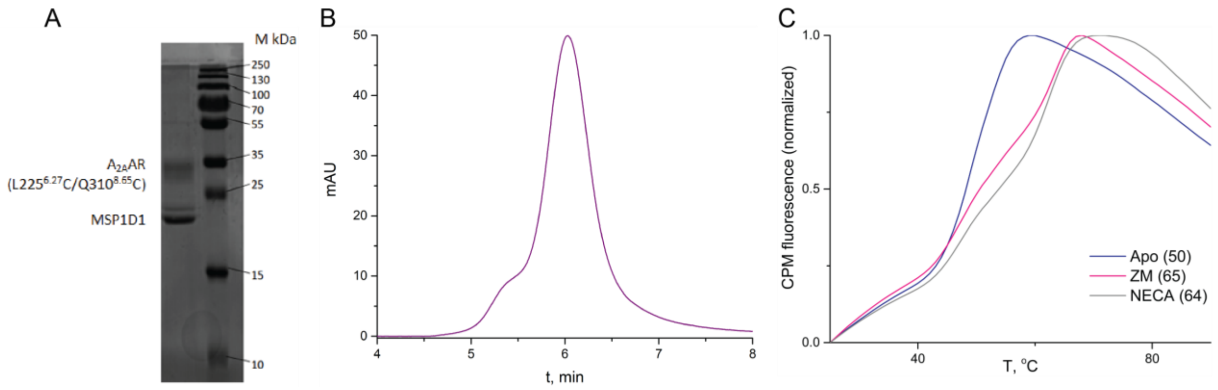

Supplementary figure 1. Characterization of the $A_{2A}AR_{L225C/Q310C}$ reconstituted in lipid nanodiscs. (**A**) SDS-PAGE analysis of the receptor reconstituted in MSP1D1 lipid nanodiscs. (**B**) Analytical SEC analysis of purified apo $A_{2A}AR_{L225C/Q310C}$ reconstituted in lipid nanodiscs. (**C**) Thermal stability assay using CPM (7-Diethylamino-3-(4'-Maleimidylphenyl)-4-Methylcoumarin) fluorescence of $A_{2A}AR_{L225C/Q310C}$ reconstituted in lipid nanodiscs in apo form, and in complex with ligands: antagonist ZM241385, and agonist NECA. Melting temperature is shown in brackets in °C.

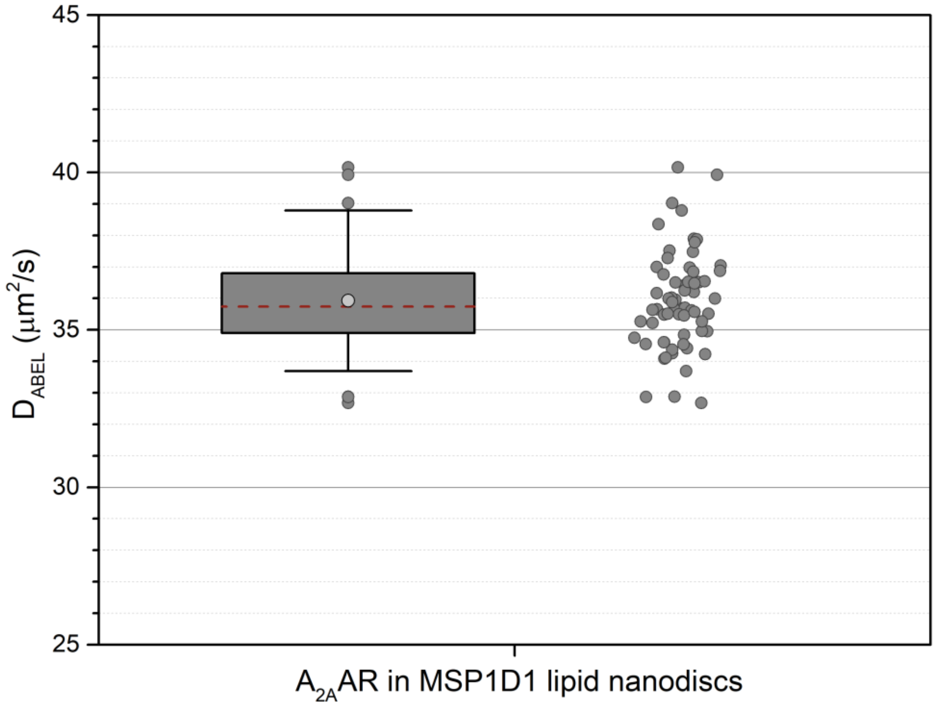

Supplementary figure 2. The distribution of diffusion coefficients of $A_{2A}AR$ in lipid nanodiscs. The diffusion coefficients were determined using the ABEL trap software (see section "Diffusion Analysis" in Methods). 64 individual $A_{2A}AR$ reconstituted in MSP1D1 lipid nanodiscs were analyzed. On the left, the box plot shows the 25-to-75 percentage data range as the grey box. The whiskers range from 5-to-95 percent, and few outliers are shown as individual data points. The mean diffusion coefficient was D_{mean} = 35.9 µm²/s (light grey dot withing the box) and the median was D_{median} = 35.7 µm²/s (red dashed line within the box). The individual data points are shown on the right.

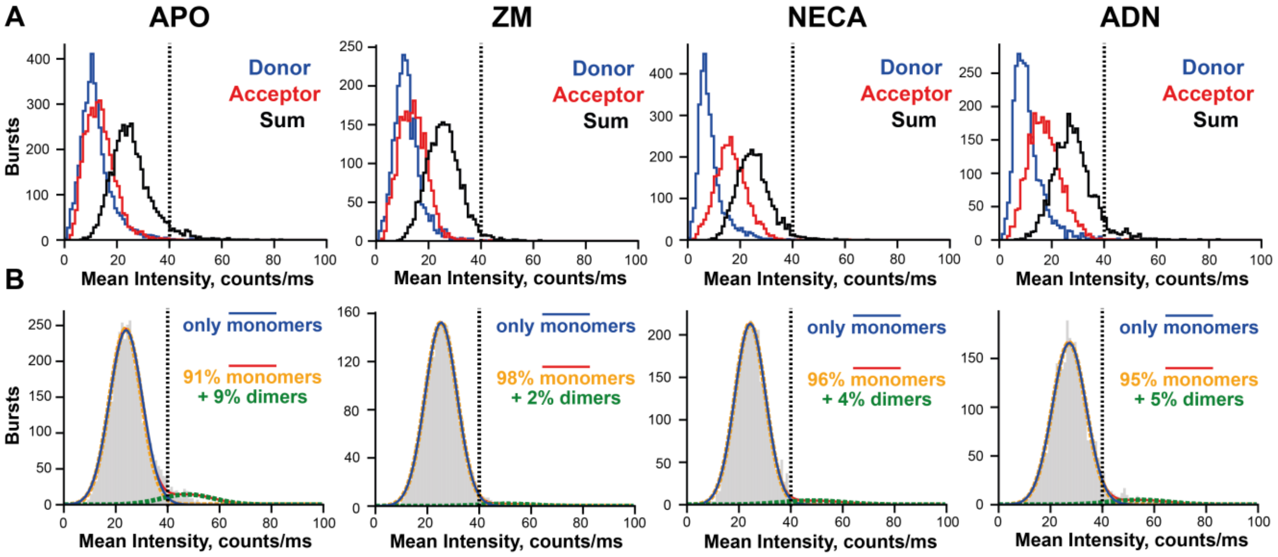

Supplementary figure 3. Analysis of fluorescence intensities of individual apo and ligand-bound A_{2A}AR molecules. (A) Histograms for fluorescence intensities in two channels and their sum, all averaged over the observation times of individual ABEL-trapped receptors. All detected and manually marked bursts, including those shorter than 100 ms, contributed to the histograms. **(B)** One-Gaussian (blue lines, "only monomers") and two-Gaussian (red lines) fits of the histograms for the sum intensity in two channels averaged over the observation times (grey stairs). Individual components of two-Gaussian fits have 2:1 intensity ratio; higher and lower intensities correspond to "dimer" (green lines) and "monomer" (orange lines) fractions, respectively. The fitting parameters are given in Table S1. The threshold of 40 photon counts per millisecond (dashed black line) was used to exclude potential oligomers and aggregates in the subsequent analyses.

	**Monomers only**	**Monomers + dimers**			
	I monomers, counts/ms	F monomers, %	F dimers, %	I monomers, counts/ms	I dimers, counts/ms
APO	24±6	91	9	24±6	48±10
ZM	25±6	98	2	25±6	51±10
NECA	24±6	96	4	24±6	49±12
ADN	28±7	95	5	27±7	54±11

Supplementary table 1. Fitting parameters of fluorescence intensity distributions for apo and ligand-bound A_{2A}AR. Fluorescence intensities in individual A_{2A}AR molecules were summed over donor and acceptor channels and averaged over the molecule's observation times. In one-Gaussian ("Monomer only") and two-Gaussian ("Monomers + dimers") fits, the intensity values (*I,* counts per millisecond) for individual components are given as mean ± SD. In the two-Gaussian fit, individual components have 2:1 intensity ratio; higher and lower intensities correspond to "dimer" (green lines) and "monomer" (orange lines) fractions, respectively. The fractions of molecules in each component (*F*, %) are also given.

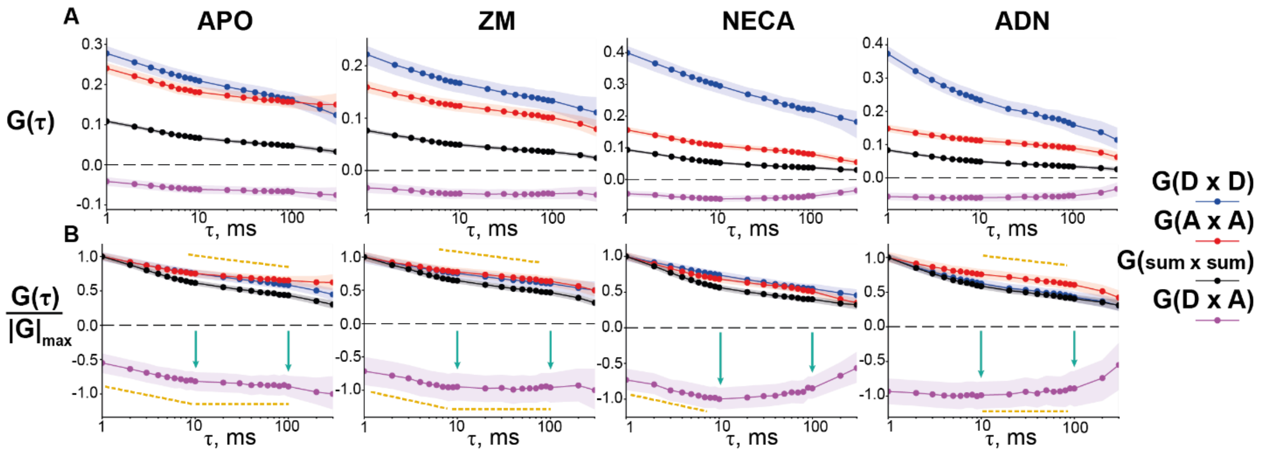

Supplementary figure 4. The correlation functions for the fluorescence intensities of donor and acceptor fluorophores in apo and ligand-bound A_{2A}AR. Autocorrelation function for donor (G_{DxD}) and acceptor (G_{AxA}) intensities, their cross-correlation function (G_{DxA}), and autocorrelation function for the total intensity in two color channels ($G_{sumxsum}$) are plotted against time lag τ. The curves are shown without normalization (A) and after normalization to the largest absolute deviation from zero (B). The 95% confidence intervals were obtained via statistical bootstrapping (shaded colored areas)[1]. Overall anticorrelation between donor and acceptor intensities and FRET-independent trends in the data are highlighted with green arrows and yellow lines, respectively (see main text for more details).

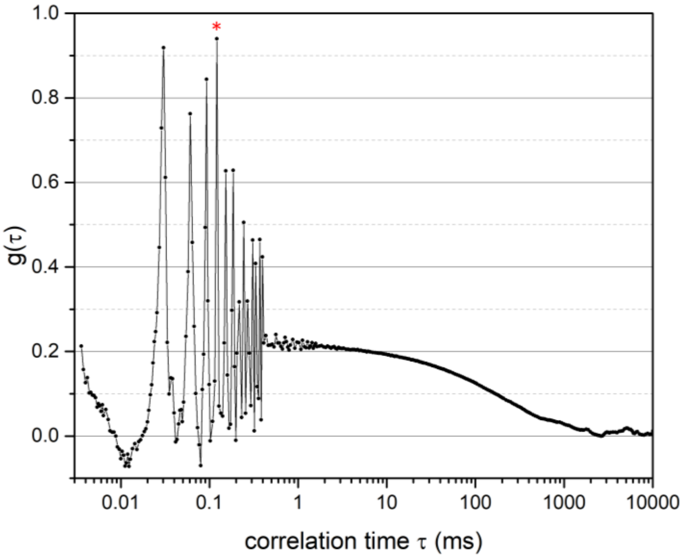

Supplementary figure 5. Normalized intensity autocorrelation function calculated from a 5-min time-correlated single photon counting (TCSPC) recording of the labeled apo A_{2A}AR in lipid nanodiscs in the working ABEL trap. Photon counts from FRET donor and acceptor fluorophores were combined for calculating the FCS curve. The photon-by-photon correlation curve was calculated as described in Zarrabi et al.[2]. The decrease in the correlation at larger delay times τ reflects diffusional escape of the receptors from the trapping region and photobleaching of the labels (donors and acceptors). The highest correlation peak at 125 µs (marked with a red star *) corresponds to the full repetition time of the laser focus pattern of the ABEL trap set to 32 points in a Knight's tour with 8 kHz repetition rate for the full pattern.

Supplementary references

1. Efron, B. & Tibshirani, R. J. *An Introduction to the Bootstrap*. (Chapman and Hall/CRC, New York, 1994). doi:10.1201/9780429246593.
2. Zarrabi, N. *et al.* Asymmetry of rotational catalysis of single membrane-bound F0F1-ATP synthase. *Proc. SPIE* **5699**, 175–188 (2005).
