# Supplementary material for: ABEL-FRET bridges the timescale gap in single-molecule measurements of the structural dynamics in the A2A adenosine receptor
Source: Commun Chem. 2026 Mar 9;9:114. doi: 10.1038/s42004-026-01941-8 (PMC12972066; doi:10.1038/s42004-026-01941-8)
Supplement: Supplementary file 2 — Reporting requirements for life sciences research [file 42004_2026_1941_MOESM2_ESM.pdf]

nature portfolio

Corresponding author(s): Michael Börsch and Thomas Gesch

Last updated by author(s): Jan 20, 2026

Reporting Summary

Nature Portfolio wishes to improve the reproducibility of the work that we publish. This form provides structure for consistency and transparency in reporting. For further information on Nature Portfolio policies, see our Editorial Policies and the Editorial Policy Checklist.

Statistics

For all statistical analyses, confirm that the following items are present in the figure legend, table legend, main text, or Methods section.

n/a	Confirmed	
☐	☒	The exact sample size (n) for each experimental group/condition, given as a discrete number and unit of measurement
☐	☒	A statement on whether measurements were taken from distinct samples or whether the same sample was measured repeatedly
☒	☐	The statistical test(s) used AND whether they are one- or two-sided<br>*Only common tests should be described solely by name; describe more complex techniques in the Methods section.*
☒	☐	A description of all covariates tested
☒	☐	A description of any assumptions or corrections, such as tests of normality and adjustment for multiple comparisons
☒	☐	A full description of the statistical parameters including central tendency (e.g. means) or other basic estimates (e.g. regression coefficient) AND variation (e.g. standard deviation) or associated estimates of uncertainty (e.g. confidence intervals)
☒	☐	For null hypothesis testing, the test statistic (e.g. F, t, r) with confidence intervals, effect sizes, degrees of freedom and P value noted<br>*Give P values as exact values whenever suitable.*
☒	☐	For Bayesian analysis, information on the choice of priors and Markov chain Monte Carlo settings
☒	☐	For hierarchical and complex designs, identification of the appropriate level for tests and full reporting of outcomes
☒	☐	Estimates of effect sizes (e.g. Cohen's d, Pearson's r), indicating how they were calculated

Our web collection on statistics for biologists contains articles on many of the points above.

Software and code

Policy information about availability of computer code

Data collection	The ABEL trap control software (Labview, National Instruments) was provided by A. Fields and A. E. Cohen and used with minor modifications to match our hardware configuration and to implement the Knight's tour pattern. The original software is described in ref. 40. Commercial SPCM software (version 8.9; Becker&Hickl) was used for smFRET data collection.
Data analysis	"Burst Analyzer" software (version 3.4.1; Becker & Hickl) was used for visualization of raw data and marking fluorescence bursts. Further analysis was performed via custom Python scripts available in Zenodo with the identifier DOI: 10.5281/zenodo.16091080.

For manuscripts utilizing custom algorithms or software that are central to the research but not yet described in published literature, software must be made available to editors and reviewers. We strongly encourage code deposition in a community repository (e.g. GitHub). See the Nature Portfolio guidelines for submitting code & software for further information.

Data

Policy information about availability of data

All manuscripts must include a data availability statement. This statement should provide the following information, where applicable:

- Accession codes, unique identifiers, or web links for publicly available datasets
- A description of any restrictions on data availability
- For clinical datasets or third party data, please ensure that the statement adheres to our policy

All data supporting this study's findings are available upon request from the corresponding author(s). The burst data from ABEL-FRET experiments are available in Zenodo with the identifier DOI: 10.5281/zenodo.16091080

Research involving human participants, their data, or biological material

Policy information about studies with human participants or human data. See also policy information about sex, gender (identity/presentation), and sexual orientation and race, ethnicity and racism.

Reporting on sex and gender	n.a. (no human participants in the study)
Reporting on race, ethnicity, or other socially relevant groupings	n.a. (no human participants in the study)
Population characteristics	n.a. (no human participants in the study)
Recruitment	n.a. (no human participants in the study)
Ethics oversight	n.a. (no human participants in the study)

Note that full information on the approval of the study protocol must also be provided in the manuscript.

Field-specific reporting

Please select the one below that is the best fit for your research. If you are not sure, read the appropriate sections before making your selection.

☒ Life sciences ☐ Behavioural & social sciences ☐ Ecological, evolutionary & environmental sciences

For a reference copy of the document with all sections, see nature.com/documents/nr-reporting-summary-flat.pdf

Life sciences study design

All studies must disclose on these points even when the disclosure is negative.

Sample size	No statistical methods were used to determine sample size. The sample size varied among apo and ligand-bound conditions due to variable quality of the PDMS-based disposable microfluidic chambers. The arbitrary threshold of > 1000 bursts/samples was used for ABEL-FRET data collection to allow the plotting of the PR-distribution and further analysis.
Data exclusions	Intensity- and duration-based thresholds were applied after manual marking of fluorescent bursts. Criteria for marking and thresholding are specified in Methods. The non-thresholded dataset is provided.
Replication	For each apo or ligand-bound condition, at least three ABEL-FRET recordings were performed, each with new disposable microfluidic chamber and new protein aliquot from the same purification and labeling batch. For each conditions, measurements were performed on at least two days. The cumulative data from all recordings are presented in the manuscript. The data from individual measurements were qualitatively similar.
Randomization	Randmization was not applicable we used identical protein samples (different aliquotes from the same protein batch) for each measurement with various ligands. Groups are not relevant to this study.
Blinding	The investigators were not blinded during data collection or during analysis.

Reporting for specific materials, systems and methods

We require information from authors about some types of materials, experimental systems and methods used in many studies. Here, indicate whether each material, system or method listed is relevant to your study. If you are not sure if a list item applies to your research, read the appropriate section before selecting a response.

Materials & experimental systems

n/a | Involved in the study

- [x] [ ] Antibodies
- [x] [ ] Eukaryotic cell lines
- [x] [ ] Palaeontology and archaeology
- [x] [ ] Animals and other organisms
- [x] [ ] Clinical data
- [x] [ ] Dual use research of concern
- [x] [ ] Plants

Methods

n/a | Involved in the study

- [x] [ ] ChIP-seq
- [x] [ ] Flow cytometry
- [x] [ ] MRI-based neuroimaging

Plants

Seed stocks	n.a.
Novel plant genotypes	n.a.
Authentication	n.a.

Jan. 20, 2026

M. [illegible]

T. [illegible]
